# LncRNA-GAS5 induces PTEN expression through inhibiting miR-103 in endometrial cancer cells

**DOI:** 10.1186/s12929-015-0213-4

**Published:** 2015-10-29

**Authors:** Chen Guo, Wei-qi Song, Ping Sun, Lian Jin, Hong-yan Dai

**Affiliations:** Department of Obstetrics & Gynaecology, Affiliated Hospital of inner Mongolia University For The Nationalities, Huolinhe Avenue East NO. 1742, Tongliao, 028000 China

**Keywords:** LncRNA-GAS5, PTEN, miR-103, Endometrial cancer, Apoptosis

## Abstract

**Background:**

Growth arrest-specific 5 (GAS5) was reported to be implicated and aberrantly express in multiple cancers. However, the expression and mechanism of action of GAS5 were largely poor understood in endometrial carcinoma.

**Results:**

According to the result of real-time reverse-transcriptase polymerase chain reaction (RT-PCR) and flow cytometry analysis, we identified that GAS5 was down-regulated in endometrial cancer cells and stimulated the apoptosis of endometrial cancer cells. To investigate the expression of GAS5, PTEN and miR-103, RT-PCR was performed. And we found that the expression of PTEN was up-regulated when endometrial cancer cells overexpressed GAS5. The prediction of bioinformatics online revealed that GAS5 could bind to miR-103, which was further found to be regulated by GAS5. Finally, we found that miR-103 mimic could decrease the mRNA and protein levels of PTEN through luciferase reporter assay and western blotting, and GAS5 plasmid may reverse this regulation effect in endometrial cancer cells.

**Conclusion:**

In summary, we demonstrate that GAS5 acts as an tumor suppressor lncRNA in endometrial cancer. Through inhibiting the expression of miR-103, GAS5 significantly enhanced the expression of PTEN to promote cancer cell apoptosis, and, thus, could be an important mediator in the pathogenesis of endometrial cancer.

## Background

Endometrial carcinoma, one of the most frequent gynecologic malignancy, is the fourth most common cancer of women in the United States [26]. Despite its prevalence, the molecular mechanisms of endometrial carcinogenesis have been poorly understood. Recently, more attention have been focused on long non-coding RNAs (lncRNAs), which was shown to regulate many key biological processes [13]. Mounting evidence indicated that the aberrant expression of some lncRNAs might play an important functional role in cancer biology [8]. LncRNAs can act as proto-oncogenes (e.g., HOTAIR) or tumor suppressor genes (e.g., GAS5 (growth arrest-specific transcript 5)) in tumorigenesis [11, 20]. GAS5 was identified using a functional screen through its ability to suppress apoptosis in a mouse thymoma cell line [5]. Several lines of evidence indicated that GAS5 was implicated and aberrantly expressed in multiple cancers, such as breast cancer, hepatocellular carcinoma, gastric cancer, bladder cancer and non-small-cell lung cancer (NSCLC) [6, 12, 16, 22, 24]. However, the expression and mechanism of action of GAS5 were largely poor understood in endometrial carcinoma.

The most frequent of genetic alterations in endometrioal carcinomas is phosphatase and tensin homologue (PTEN). PTEN is a tumor suppressor gene located on 10q23, and is reportedly involved in the regulation of focal adhesion, cellular migration, and tumor cell proliferation [10, 23]. In addition, PTEN mutations were identified in about 20 % of cases of endometrial hyperplasia, a precursor of endometrial carcinoma [15]. Accordingly, inactivation of PTEN is considered to be an early event in endometrial carcinogenesis [18]. Mieko Matsushima-Nishiu et al. reported that PTEN possessed the ability of inducing cell cycle arrest and apoptosis [14].

Recently, numerous microRNAs (miRNAs) have been demonstrated to promote tumorigenesis or metabolic disorders by down-regulating PTEN expression [21]. Among them, miR103 was found to promote colorectal cancer through down-regulation the expression of PTEN [7]. For miR-103, Boren et al. and Chung et al. first identified the aberrant overexpression of miR-103 in endometrial cancer [2, 4]. Moreover, DONGQI YU et al. found that miR-103 stimulated growth and invasion of endometrial cancer cell lines through post-transcriptionally down-regulating the expression of the tumor suppressor TIMP-3 [27]. In this study, we found that the prediction of bioinformatics analysis revealed the competitive inhibition of GAS5 on the expression of miR-103. On the basis of this results, we aimed to evaluate the possible involvement of GAS5 in endometrial carcinogenesis and to reveal any correlation between GAS5 and PTEN, miR-103. The results of this study may aid the understanding of the effects of GAS5 in the tumor suppression and progression of endometrial cancer.

## Methods

### Patients

The experiment protocols were approved by the Human Studies Committee at the Affiliated Hospital of inner Mongolia University For The Nationalities and informed consent was obtained from each patient prior surgery. Tissue specimens were obtained from 20 of endometrial cancer patients and 20 of uninvolved controls. The characteristic of endometrial cancer patients and controls were summarized in Table 1. Endometrial cancer samples were obtained from patients undergoing hysterectomy without preoperative chemotherapy or radiation and histologically validated for type and grade.

**Table 1:** Characteristics of endometrial cancer specimens and non-cancer specimens

	Sample no.
(a) Endometrial cancer	
Total no.	20
Median age, years (range)	56.7 (38–76)
Pathological tumor stage	
I	11 (55 %)
II	3 (15 %)
III	4 (20 %)
IV	2 (10 %)
Differentiation	
G1	5 (25 %)
G2	7 (35 %)
G3	8 (40 %)
Lymphatic metastasis	
(+)	7 (35 %)
(−)	12 (60 %)
Unknown	1 (5 %)
(b) Normal endometrium	
Total no.	20
Median age, years (range)	50 (36–75)

Normal endometrial samples were obtained from premenopausal women awaiting in vitro fertilization treatment. All of the samples were frozen in liquid nitrogen immediately after resection and stored at −80 °C until use.

### Cell lines and cell culture

The human endometrial cancer cell lines HHUA and JEC were acquired from ATCC and grown in Minimum Essential Medium Eagle (Sigma-Aldrich, UK) supplemented with 15 % of fetal bovine serum (Gibco, Darmstadt, Germany), 100 U/ml of penicillin and 100 μg/ml of streptomycin (Gibco). The cells were incubated at 37 °C in a humidified atmosphere of 5 % CO_2_.

### Construction of the plasmid vector, RNA interference by siRNA and DNA transfection

The full-length GAS5 sequence lacking a poly-A tail was synthesized according to National Center for Biotechnology Information database and sub-cloned into pcDNA3.1 (Jikai, Shanghai, China). Three siRNA-GAS5 were used to knockdown the expression of GAS5. The empty vector or the pcDNA-GAS5 vector or si-GAS5 were transfected into HHUA and JEC cells cultured in six-well plates using Lipofectamine 2000 (Invitrogen), according to the manufacturer’s instructions. Following 24 h or 48 h of transfection, the cells were harvested and processed for further analysis.

### Luciferase reporter assay

The 3′-UTR segments of PTEN mRNA were amplified by PCR from human genomic DNA and inserted into the pGL3 vector (Promega Corporation, Madison, WI, USA), designated pGL3-PTEN. The reporter plasmid (300 ng) was transfected into HHUA and JEC cells in 12-well plates with a Renilla plasmid (10 ng) using Lipofectamine 2000 (Invitrogen). At 6 h after transfection, the cells were transfected with miR-103 mimic or miR-103 mimic and pcDNA-GAS5 or the negative control oligonucleotide. The reporter assay was performed 42 h post-transfection using the Dual luciferase assay system(Promega) and the luciferase activity of each sample was normalized to the Renilla luciferase activity.

### Flow cytometry analysis of apoptosis

Apoptosis was detected by a flow cytometric analysis of Annexin V and PE staining. The pcDNA-GAS5 or empty vector-transfected HHUA and JEC cells were cultured in six-well plates for 48 h. The Annexin V-FITC versus PE assay was performed following the manufacturer’s instructions. After the double staining with Annexin V-FITC and PE, flow cytometry analysis (FACScan, BD Biosciences, San Jose, CA, USA) using the CellQuest software (BD Biosciences) was used to quantify apoptosis.

### Cell migration assay

Transwell inserts were coated with 0.1 % gelatin (Sigma). Bottom chamber was added the platelet-derived growth factor (PDGF) at 1 and 10 ng/mL dissolved in DMEM medium containing 0.1 % FBS. HHUA and JEC cells transfected with GAS5 plasmid, miR-103 mimic + GAS5 plasmid, miR-103 mimic and suspended in 100 L of DMEM containing 0.1 % BSA was added to the upper chamber. After 5 h of incubation, cells on both side of the membrane were fixed and stained with Diff-Quick staining kit. Cells on the upper side of the membrane were removed with a cotton swab.

### Western blot analysis

The protein concentrations of the total cell lysates were measured using the Micro BCA protein assay kit and were separated on SDS-PAGE (10 %) and transferred to nitrocellulose membranes. The specific antibodies for PTEN were purchased from Santa Cruz Biotechnology (Santa Cruz, CA, USA), and the antibodies for anti-GAPDH were obtained from Cell Signaling Technology (Beverly, MA, USA). GAPDH was used as a control. The membrane was incubated with the secondary antibody for 2 h. Finally, the enhanced chemiluminescence (ECL) kit were used according to the manufacturer’s instructions and to visualize the immunoreactive protein bands. The relative levels of protein expression was quantified with densitometry analysis.

### Quantitative real-time PCR

Total RNA was isolated from the cells using TRIzol reagent (Invitrogen) to obtain miRNA and mRNA. For the expression analysis of miR-103, total RNA (15 ng) was applied in TaqMan MicroRNA Assay Kit (Applied Biosystems) according to the manufacturer’s instructions. For mRNA detection, the isolated RNA was reverse transcribed into cDNA using a reverse transcription kit (Takara, Dalian, China). mRNA were quantified by qRT-PCR using SYBR Premix Ex Taq II (Perfect Real Time; TaKaRa) according to the manufacturer’s instructions. The relative expression was calculated using the ΔCT method and the expression of miR-103 was normalized using the 2^-∆∆CT^ method relative to U6-snRNA and GAS5-mRNA and PTEN-mRNA were normalized to GAPDH. The qRT-PCR assays were performed in duplicate and the data were presented as the mean ± standard error of the mean (SEM).

### Statistical analysis

Statistical analysis was performed using the SPSS 13.0 software (Chicago, IL, USA). The values are presented as the mean ± SEM. The differences/correlations between the groups were calculated using the Student’s *t*-test and analysis of variance. *P* < 0.05 was considered to indicate a statistically significant result.

## Results and discussion

### LncRNA-GAS5 was down-regulated in endometrial cancer cells and induced apoptosis of endometrial cancer cells

**Fig. 1:**
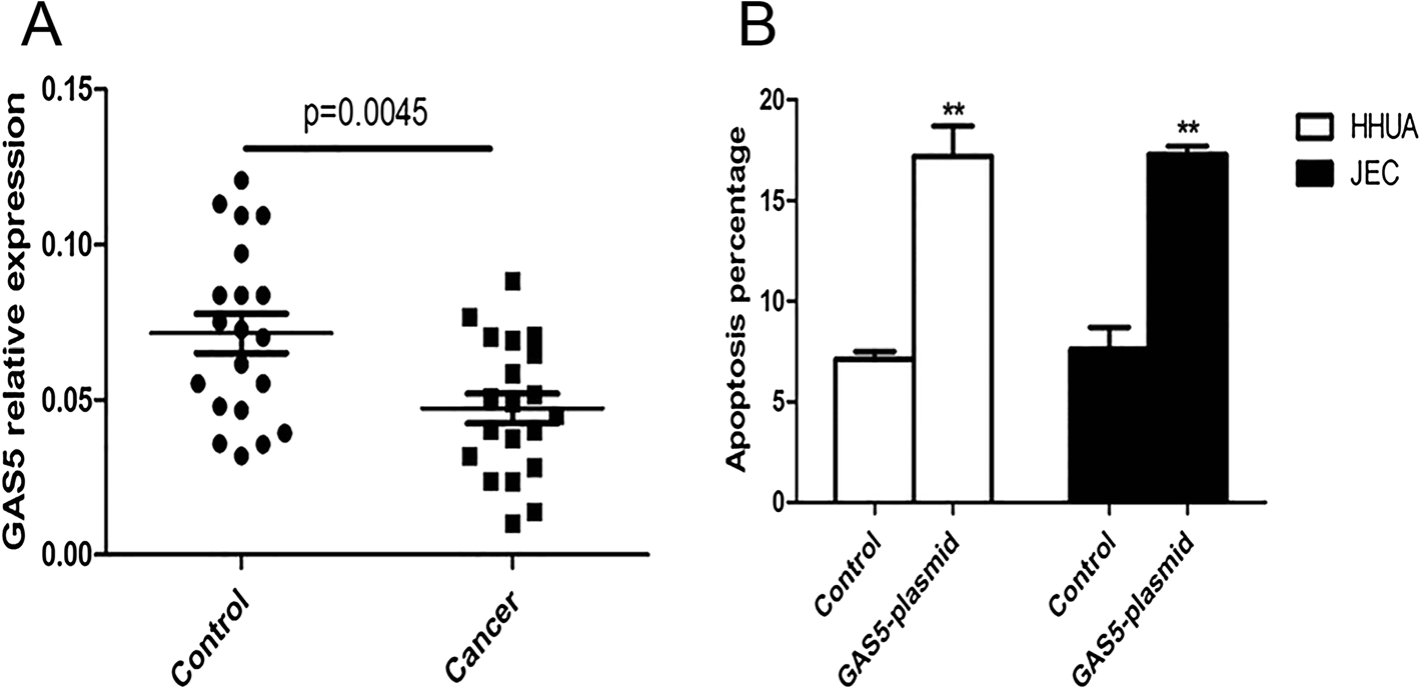
LncRNA-GAS5 was down-regulated in endometrial cancer cells and induced apoptosis of endometrial cancer cells. **a** The relative expression of LncRNA-GAS5 in tissue of endometrial cancer patients and controls. **b** The effect of LncRNA-GAS5 overexpression on apoptosis of endometrial cancer cell lines HHUA and JEC. All values are mean ± SD. **VS control, *p* < 0.01

To explore the role of lncRNA-GAS5 in endometrial cancer cells, we examined the relative expression of lncRNA-GAS5 in tissues from endometrial cancer patients and health controls with qRT-PCR. Lower mRNA level of lncRNA-GAS5 was observed in endometrial cancer patients than that of health controls (Fig. 1a). As lncRNA-GAS5 was reported to involve in apoptosis of several cancers, we detected the effect of lncRNA-GAS5 overexpression on apoptosis of endometrial cancer cell lines HHUA and JEC. After transfected the GAS5-plasmid into HHUA and JEC for 48 h, Annexin-V assay was performed to examine the percentage of apoptotic cells. It has been shown that apoptosis percentage in HHUA and JEC transfected with GAS5-plasmid were significantly higher than that in cells transfected with control plasmid (Fig. 1b). These findings indicated that the dysregulation of lncRNA-GAS5 might contribute to the development of endometrial cancer and exert tumor suppressor function.

### GAS5 regulated the expression of PTEN

**Fig. 2:**
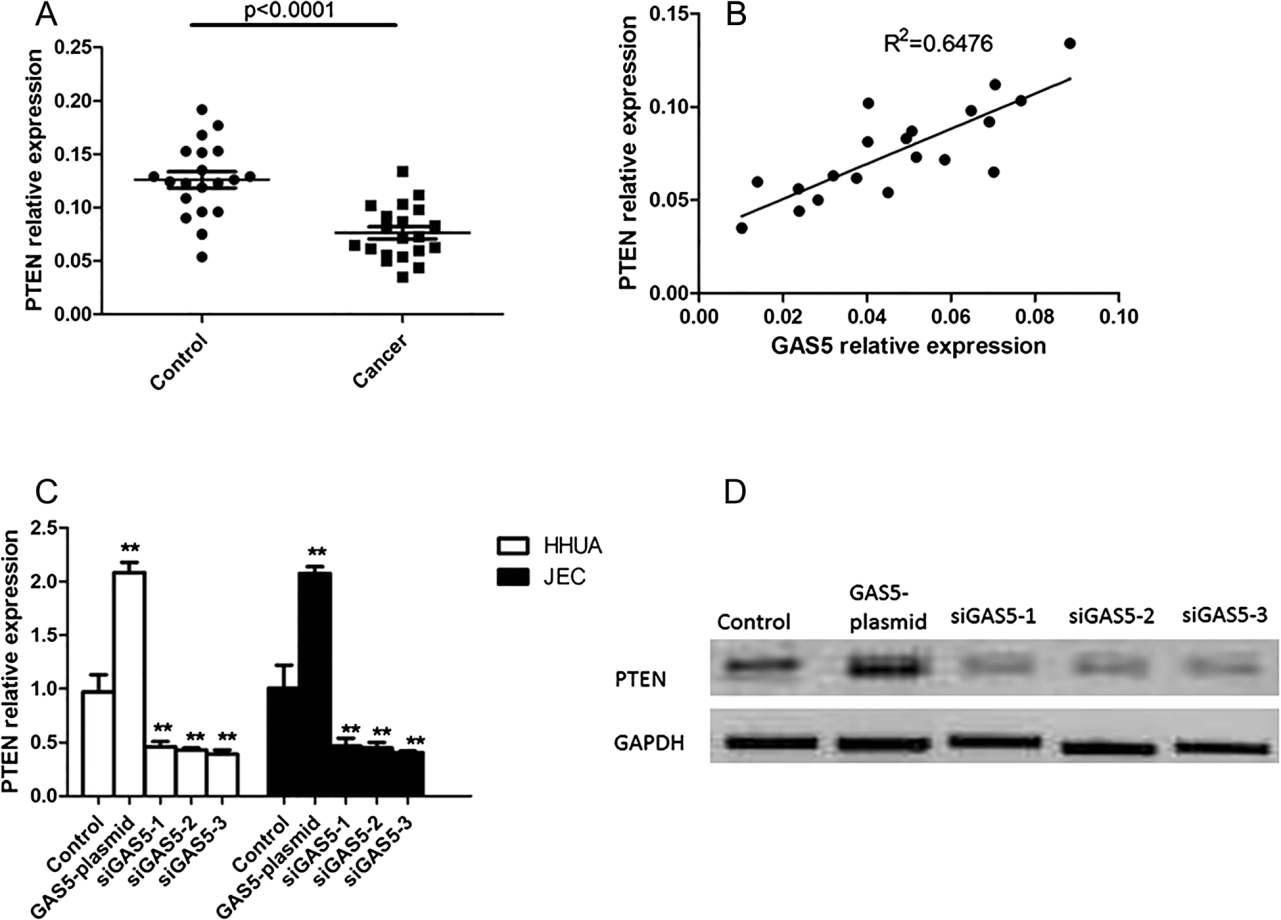
LncRNA-GAS5 regulated the expression of PTEN. **a** The relative expression of PTEN in tissue of endometrial cancer patients and controls. **b** Correlation analysis of the expression of LncRNA-GAS5 and PTEN in endometrial cancer patients. **c** The mRNA level of PTEN in HHUA and JEC cells transfected with GAS5 plasmid or si-GAS5. **d** The protein level of PTEN in HHUA cells transfected with GAS5 plasmid or si-GAS5. All values are mean ± SD. **VS control, *p* < 0.01

We further detected the expression of PTEN in tissue of endometrial cancer patients and health controls. As shown in Fig. 2a, the expression of PTEN was also down-regulated in tissues of endometrial cancer patients. To investigate the relationship between PTEN and GAS5 in endometrial cancer, correlation analysis between the expression of GAS5 and PTEN was performed. The expression of GAS5 was positively correlated with the expression of PTEN. Next, the mRNA and protein level of PTEN in HHUA and JEC cells transfected with GAS5 plasmid or si-GAS5 were determined to further elucidate the regulation of GAS5 on the expression of PTEN. It has been shown that PTEN was up-regulated in endometrial cancer cells when they were transfected with GAS5 plasmid. On the other hand, the mRNA and protein level of PTEN were reduced by the three si-GAS5 in endometrial cancer cells (Fig. 2b and c). These data suggested that GAS5 up-regulated the expression of PTEN in endometrial cancer cells.

### LncRNA-GAS5 suppressed the expression of miR-103

**Fig. 3:**
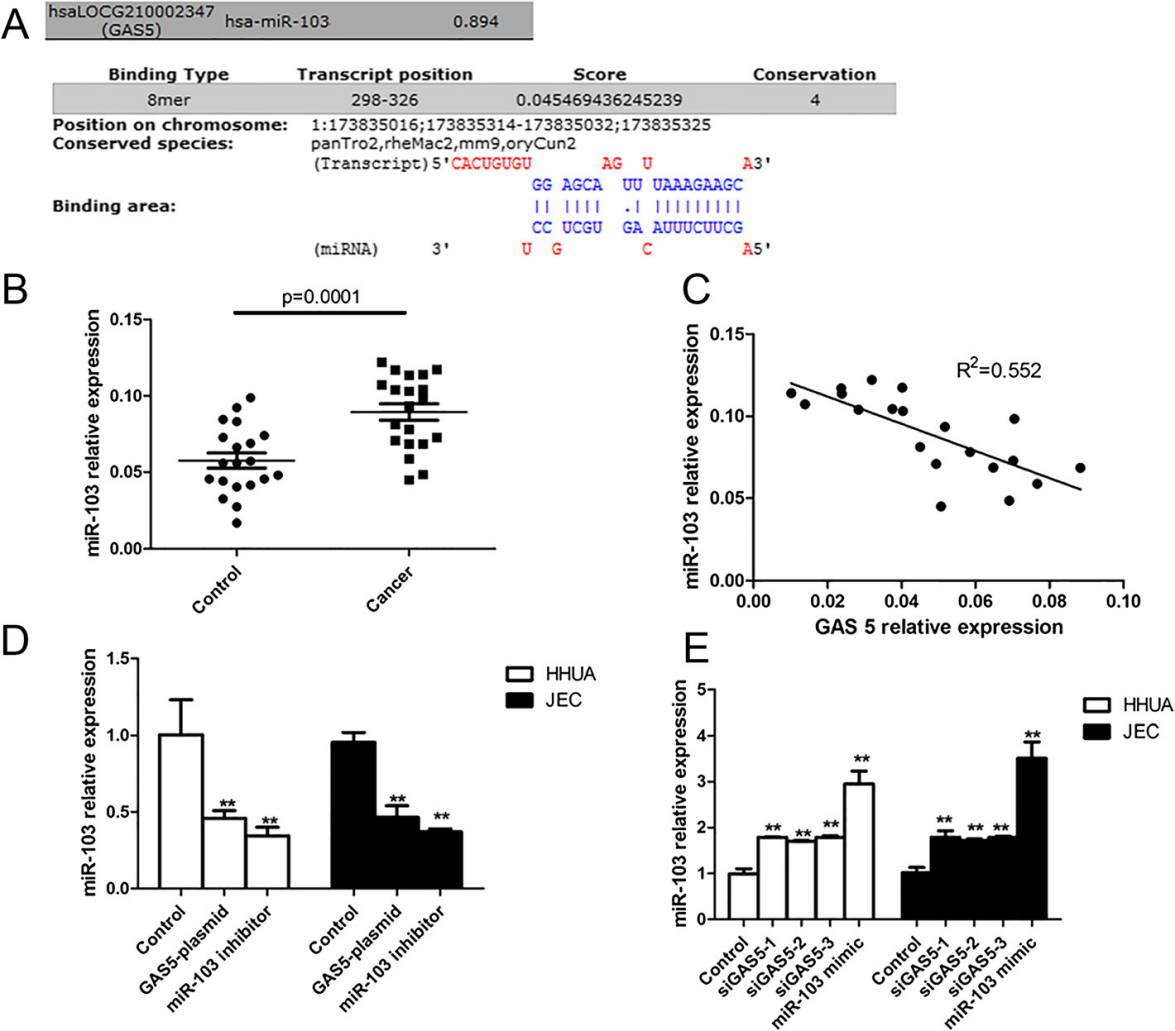
LncRNA-GAS5 suppressed the expression of miR-103. **a** Bioinformatics analysis the combination of LncRNA-GAS5 and miR-103. **b** The relative expression of miR-103 in tissue of endometrial cancer patients and controls. **c** Correlation analysis of the expression of LncRNA-GAS5 and miR-103 in endometrial cancer patients. **d** The mRNA level of miR-103 in HHUA and JEC cells transfected with GAS5 plasmid. **e** The mRNA level of miR-103 in HHUA and JEC cells transfected with si-GAS5. All values are mean ± SD. **VS control, *p* < 0.01

Through the prediction of bioinformatics analysis online, we found that GAS5 could bind to miR-103 (Fig. 3a). We further analyzed the expression of miR-103 in tissue of endometrial cancer patients and health controls. And we identified that miR-103 was substantial up-regulated in tissue of endometrial cancer patients (Fig. 3b). In addition, correlation analysis revealed that there was a negatively correlation between the expression of GAS5 and miR-103 (Fig. 3c). When HHUA and JEC cells overexpressed GAS5 through transfecting with GAS5 plasmid, we found that the expression of miR-103 was significantly decreased (Fig. 3d). While as shown in Fig. 3e, the mRNA level of miR-103 in HHUA and JEC cells transfected with si-GAS5 were significantly higher comparing to the control.

### LncRNA-GAS5 induces PTEN expression through inhibiting miR-103 in endometrial cancer cells

**Fig. 4:**
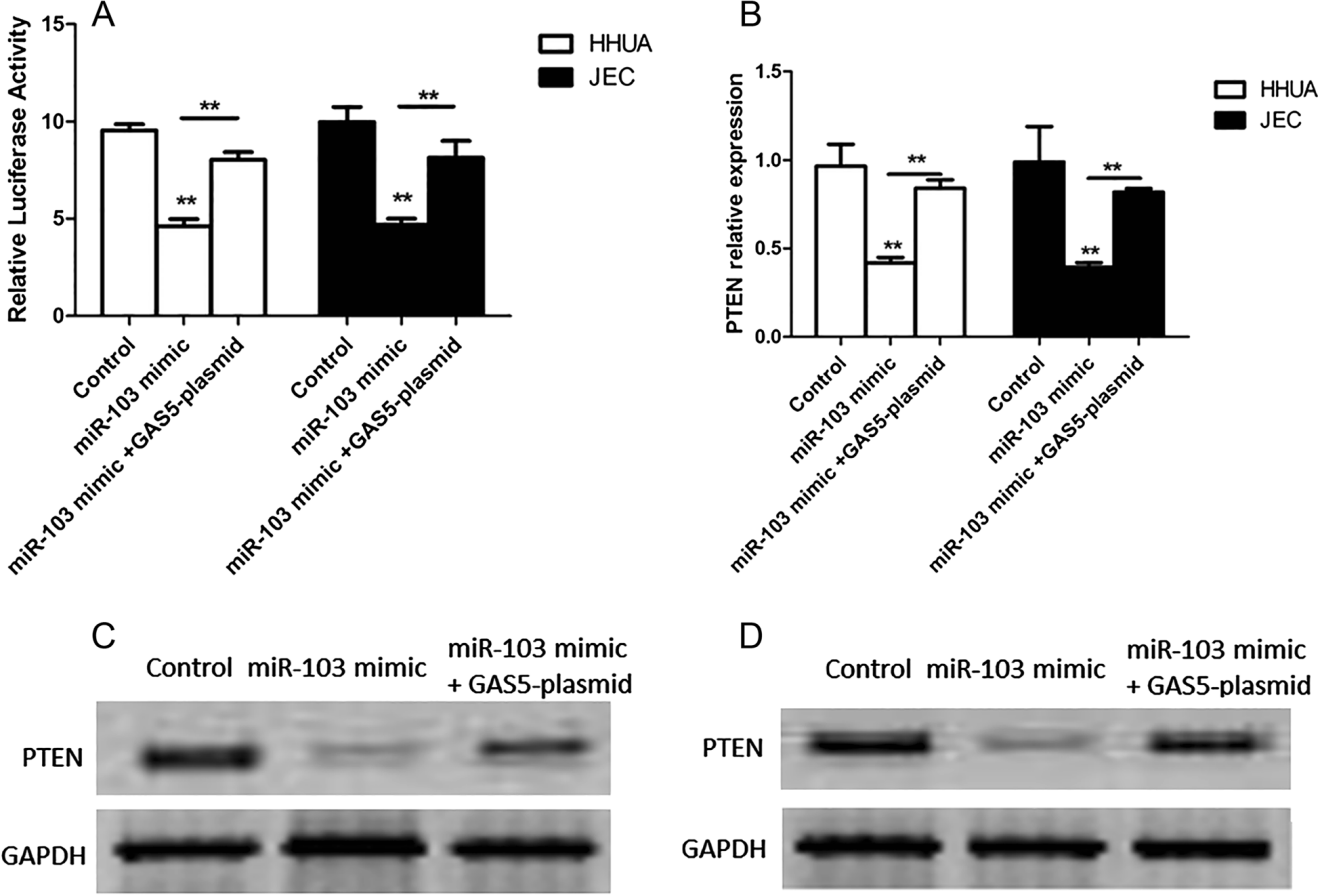
LncRNA-GAS5 induces PTEN expression through inhibiting miR-103 in endometrial cancer cells. **a** Relative luciferase activity of PTEN 3′UTR in HHUA and JEC cells transfected with miR-103 mimic + GAS5 plasmid or miR-103 mimic. **b** The mRNA level of PTEN in HHUA and JEC cells transfected with luciferase reporter vector and miR-103 mimic + GAS5 plasmid or miR-103 mimic. **c** The protein level of PTEN in HHUA cells transfected with luciferase reporter vector and miR-103 mimic + GAS5 plasmid or miR-103 mimic. **d** The protein level of PTEN in JEC cells transfected with luciferase reporter vector and miR-103 mimic + GAS5 plasmid or miR-103 mimic. All values are mean ± SD. **VS control, *p* < 0.01

To further investigate the mechanism of the regulation of PTEN and miR-103 by GAS5, HHUA and JEC cells were transfected with luciferase reporter vector containing PTEN 3′UTR and miR-103 mimic, or miR-103 mimic + GAS5 plasmid. Through measuring the luciferase activity and the mRNA level of PTEN, we found that they were both reduced in cells transfected with miR-103 mimic (Fig. 4a). Moreover, GAS5 plasmid reversed this regulation and the luciferase activity and mRNA level of PTEN were up-regulated in cells transfected with miR-103 mimic + GAS5 plasmid (Fig. 4b). Next, we detected the protein level of PTEN in above HHUA and JEC cells. As shown in Fig. 4c and d, lower protein levels of PTEN were observed in HHUA and JEC cells transfected with miR-103 mimic comparing to the control, and they were increased in HHUA and JEC cells transfected with miR-103 mimic + GAS5 plasmid. Taken together, these findings indicated that GAS5 induced PTEN expression through inhibiting miR-103 in endometrial cancer cells.

### The effect of PTEN knockdown or miR-103 expression on the apoptosis and migration of endometrial cancer cells induced by GAS5

**Fig. 5:**
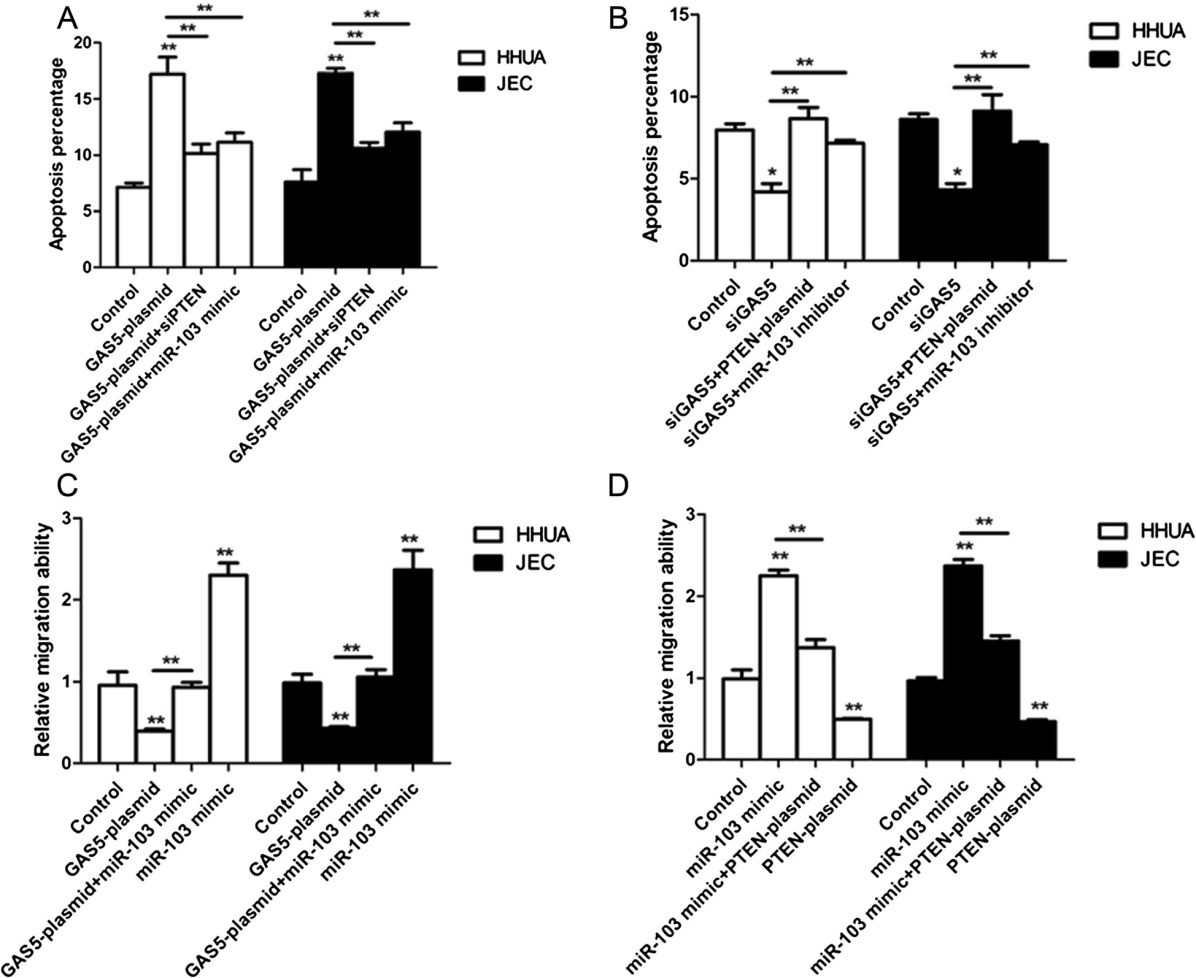
The effect of PTEN knockdown or miR-103 expression on the apoptosis and migration of endometrial cancer cells induced by GAS5. **a** The apoptosis percentage of HHUA and JEC cells transfected with control, GAS5 plasmid, GAS5 plasmid + si-PTEN and GAS5 plasmid + miR-103 mimic. **b** The apoptosis percentage of HHUA and JEC cells transfected with control, si-GAS5, si-GAS5 + PTEN plasmid and si-GAS5 + miR-103 inhibitor. **c** The relative migration ability of HHUA and JEC cells transfected with control, GAS5 plasmid, miR-103 mimic + GAS5 plasmid, miR-103 mimic. **d** The relative migration ability of HHUA and JEC cells transfected with control, miR-103 mimic, miR-103 mimic + PTEN plasmid and PTEN plasmid. All values are mean ± SD. **VS control, *p* < 0.01

To investigate whether PTEN knockdown or miR-103 expression can rescue the apoptosis in GAS5 overexpressing cells, HHUA and JEC cells were transfected with control, GAS5 plasmid, GAS5 plasmid + si-PTEN and GAS5 plasmid + miR-103 mimic. As shown in Fig. 5a, PTEN knockdown and miR-103 overexpression highly inhibited the apoptosis of GAS5 overexpressing HHUA and JEC cells. Accordingly, PTEN overexpression and miR-103 inhibitor reversed the apoptosis of HHUA and JEC cells induced by si-GAS5 (Fig. 5b). We also detect the effect of GAS5 plasmid, miR-103 mimic + GAS5 plasmid, miR-103 mimic and the comparative control on the migration abilities of the HHUA and JEC cells. As shown in Fig. 5c, GAS5 plasmid inhibited the migration of the HHUA and JEC cells and miR-103 mimic could promote this migration ability. In addition, miR-103 mimic reversed the effect of GAS5 plasmid on the migration ability. To confirm the interaction of miR-103 and PTEN in tumorigenic pathway, the migration abilities of the cancer cells transfected with control, miR-103 mimic, miR-103 mimic + PTEN plasmid and PTEN plasmid were detected. As shown in Fig. 5d, PTEN plasmid inhibited the migration abilities of the cancer cells and PTEN overexpression reversed the effect of miR-103 mimic on migration abilities.

## Discussion

In this study, we identified that GAS5 was down-regulated in endometrial cancer cells and stimulated the apoptosis of endometrial cancer cells. In addition, the expression of PTEN was up-regulated when endometrial cancer cells overexpressed GAS5. The prediction of bioinformatics online revealed that GAS5 could bind to miR-103. Through transfecting GAS5 plasmid or si-GAS5 into endometrial cancer cells, the mRNA level of miR-103 were significantly decreased or increased, respectively. We found that miR-103 mimic could decrease the mRNA and protein levels of PTEN, and GAS5 plasmid may reverse this regulation effect in endometrial cancer cells. Finally, the tumorigenic effect and migration abilities of the cancer cells were compared between GAS5, miR-103 and PTEN. Taken together, we found that GAS5 involved in the up-regulation of PTEN in endometrial cancer cells through inhibiting the expression of miR-103.

To date, numerous lncRNA genes have been identified in the human genome and several of them were found to involve in the regulation of various molecular and cellular functions [25]. Specially, GAS5 has been supposed to play a tumor-suppressive role and was down-regulated in many cancers [12]. The down-regulation of GAS5 expression is thought to contribute to tumor formation and to affect proliferation and apoptosis [17]. M Mourtada-Maarabouni et al. identified GAS5 as critical to the control of mammalian apoptosis and cell population growth in breast cancer cell lines [16]. In this study, we also found that the mRNA level of GAS5 was reduced in endometrial cancer cells. In addition, Annexin-V assay showed that GAS5 promoted the apoptosis of HHUA and JEC.

As PTEN was the most commonly mutated gene identified in endometrial carcinoma, we investigated the effect of GAS5 on the expression of PTEN. The results of correlation analysis showed that GAS5 was positively correlated with the expression of PTEN. In addition, the expression of PTEN was increased in endometrial cancer cells transfected with GAS5 plasmid. PTEN has dual protein and lipid phosphatase activity. An increasing body of evidence shows that PTEN functions as a tumour suppressor gene in some tissues, and its tumour suppressor activity is dependent on its lipid phosphatase activity, which negatively regulates the PI3K-AKT-mTOR pathway [21]. PTEN also mediated the up-regulation of proapoptotic mechanisms involving AKT-dependent mechanisms and the down-regulation of antiapoptotic mechanisms through Bcl-2 [1]. In 2000, the decreased PTEN expression or function was reported to be a marker of the earliest endometrial precancers [18]. Later, Helga B. Salvesen et al. reported on PTEN protein expression in a large and population-based series of patients with endometrial carcinoma and suggested that loss of PTEN protein staining was relatively frequent in endometrial carcinoma [19].

The identification of the molecular mechanisms that control the expression of PTEN is now being elucidated. MiRNAs have been demonstrated to regulate the expression of PTEN in tumorigenesis or metabolic disorders [21]. Here, we found the regulative function of miR-103 on the expression of PTEN in endometrial cancer cells, which was consistent with the report of Li Geng et al. [7]. On the basis of bioinformatics prediction, we identified that GAS5 could bind to miR-103, which was further found to be regulated by GAS5. In addition, GAS5 might reverse the regulation effect of miR-103 on PTEN in endometrial cancer cells. Being contrary to the tumour suppressor function of GAS5 and PTEN, miR-103 was found to stimulate growth and invasion in endometrial cancer cell lines. MiR-103 is also reported to relate with several human cancers. Recent reports showed miR-103 was expressed in colorectal cancer as an oncogenic miRNA by targeting DAPK, KLF4 and PER3 [3, 9].

## Conclusions

In summary, we demonstrate that GAS5 acts as an tumour suppressor lncRNA in endometrial cancer. Through inhibiting the expression of miR-103, GAS5 significantly enhanced the expression of PTEN to promote cancer cell apoptosis, and, thus, could be an important mediator in the pathogenesis of endometrial cancer. Manipulation of GAS5 axis expression might represent a novel potential therapeutic target for endometrial cancer treatment.

## Abbreviations

GAS5: Growth arrest-specific 5
lncRNAs: Long non-coding RNAs
NSCLC: Non-small-cell lung cancer
PTEN: Phosphatase and tensin homologue
